# On the Use of Glycerine in the Treatment of Certain Forms of Deafness

**Published:** 1852-08

**Authors:** Thomas Wakley

**Affiliations:** F.R.C.S., Eng., Surgeon to the Royal Free Hospital, London


From the London Lancet.

*On the Use of Glycerine in the Treatment of Certain Forms of Deafness.* By THOMAS WAKLEY, Esq., F.R.C.S., Eng., Surgeon to the Royal Free Hospital, London.

THE class of cases to which I would draw your attention in this report, are those of *cuticular* or *epithelial thickening* of the meatus, either *partial*, affecting the membrane of the tympanum, or *complete*, being continued over the entire auditory cul-de-sac. There is a greater or less degree of deafness, corresponding with the amount of thickening; cessation of the secretion of cerumen; frequently tinnitus, or a "singing and hissing sensation" in the ears, and tickling irritation of the meatus. The causes are, constitutional predisposition, advanced age, chronic inflammation, long continued discharge following erruptive fevers and the application of escharotics and irritants. Amongst the latter, I would mention oily preparations, the globules of which adhere to the sides of the meatus or membrana tympani, and become rancid, thus producing a very frequent cause of inflammation. Upon examination of the affected ear, we find the meatus shining and inelastic, of a pearly whiteness, the membrana tympani either clouded or streaked, sometimes having small elevations upon it. The meatus is quite dry, the cerumenous glands being choked up by the epithelial growth.

The mode of application of the glycerine, when treating this state of the ear, is as follows:—The meatus is well cleansed with tepid water, and then dried by means of the forceps and cotton. Glycerine is now poured into the meatus, and a plug of gutta percha, softened in boiling water, made to fit the external opening; this takes the exact form of the ear, becomes hard, and effectually prevents either the entrance of atmospheric air or the exit of the glycerine. The ear should be examined daily and the same process repeated. The lining membrane can be examined with a blunt silver probe, passed gently through the speculum auris, to ascertain the effect of the glycerine upon the cuticular thickening. The meatus will gradually lose its shining pearly appearance, and softened pieces will fall off, and can be removed either by the forceps, or gentle syringing. The practitioner should never attempt to tear them away, but allow them to come away by the means just stated. The treatment occupies ordinarily from two to four weeks, and is generally without any pain or inconvenience of any kind to the patient, and the results, in some cases, have been very gratifying. In the after treatment the patients are directed to moisten the auditory canal at least once a week with glycerine, applied by means of a camel hair brush; this will generally prevent a recurrence of the cuticular thickening.

The *modus operandi* is simple enough—the glycerine being kept continually in contact with the part, acts mechanically, either absorbing or penetrating the epithelial coating, and separating the individual particles.

With respect to the permanence of the relief—some cases always require the presence of glycerine as the best known substitute for the natural secretion of the aural membrane. The frequent introduction of the glycerine tends to restore the external meatus to a healthy condition, and fit it for the healthy transmission of sound.

